# Room‐Temperature Band‐Aligned Infrared Heterostructures for Integrable Sensing and Communication

**DOI:** 10.1002/advs.202401716

**Published:** 2024-06-05

**Authors:** Kening Xiao, Shi Zhang, Kaixuan Zhang, Libo Zhang, Yuanfeng Wen, Shijian Tian, Yunlong Xiao, Chaofan Shi, Shicong Hou, Changlong Liu, Li Han, Jiale He, Weiwei Tang, Guanhai Li, Lin Wang, Xiaoshuang Chen

**Affiliations:** ^1^ College of Physics and Optoelectronic Engineering Hangzhou Institute for Advanced Study University of Chinese Academy of Sciences No. 1, Sub‐Lane Xiangshan, Xihu District Hangzhou 310024 China; ^2^ State Key Laboratory of Infrared Physics Shanghai Institute of Technical Physics Chinese Academy of Sciences 500 Yu‐Tian Road Shanghai 200083 China; ^3^ School of Physical Science and Technology ShanghaiTech University Shanghai 201210 China; ^4^ University of Chinese Academy of Sciences No. 19A Yuquan Road Beijing 100049 China

**Keywords:** optical communication, topological insulator, type‐II van der Waals integration heterojunction, wide spectral

## Abstract

The demand for miniaturized and integrated multifunctional devices drives the progression of high‐performance infrared photodetectors for diverse applications, including remote sensing, air defense, and communications, among others. Nonetheless, infrared photodetectors that rely solely on single low‐dimensional materials often face challenges due to the limited absorption cross‐section and suboptimal carrier mobility, which can impair sensitivity and prolong response times. Here, through experimental validation is demonstrated, precise control over energy band alignment in a type‐II van der Waals heterojunction, comprising vertically stacked 2D Ta_2_NiSe_5_ and the topological insulator Bi_2_Se_3_, where the configuration enables polarization‐sensitive, wide‐spectral‐range photodetection. Experimental evaluations at room temperature reveal that the device exhibits a self‐powered responsivity of 0.48 A·W^−1^, a specific directivity of 3.8 × 10^11^ cm·Hz^1/2^·W^−1^, a response time of 151 µs, and a polarization ratio of 2.83. The stable and rapid photoresponse of the device underpins the utility in infrared‐coded communication and dual‐channel imaging, showing the substantial potential of the detector. These findings articulate a systematic approach to developing miniaturized, multifunctional room‐temperature infrared detectors with superior performance metrics and enhanced capabilities for multi‐information acquisition.

## Graphical abstract

This study validates precise energy band alignment control in a type‐II van der Waals heterojunction, composing 2D Ta_2_NiSe_5_ with topological insulator Bi_2_Se_3_, enabling polarization‐sensitive, extensive‐spectral‐range photodetection. Room temperature tests show the heterojunction boasts a self‐powered responsivity of 0.48 A·W^−1^, a specific directivity of 3.8 × 10^11^ Jones, and a response time of 151 µs, highlighting its promise for infrared communication and dual‐imaging applications.

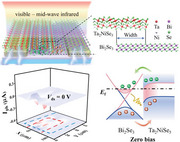

## 1 Introduction

The burgeoning demand for miniaturized and portable photosensitive devices, capable of self‐powered operation at room temperature, has catalyzed significant scientific interest in developing innovative technologies for applications such as remote sensing, target identification, gas sensing, imaging, and free‐space optical communications.^[^ ^1^, ^2^, ^3^, ^4^, ^5^ ^]^ Currently, the commercial infrared photodetectors, which typically employ conventional narrow bandgap semiconductors such as In*_x_* Ga_1‐_ *_x_* As,^[^ ^6^ ^]^ InSb,^[^ ^7^ ^]^ and Hg_1‐_ *_x_* Cd*_x_* Te,^[^ ^8^ ^]^ suffer from stringent growth conditions required to tune bandgap to match the operational range from the short to long‐wave infrared spectra. Additionally, such detectors often require cryogenic cooling to reduce thermal noise and biased operation for charge separation, which increases both size and energy consumption of the detectors, thus limiting broader applicability.^[^ ^9^, ^10^ ^]^ The growing intersection of the materials science and nanotechnology research is driving the exploration of low‐dimensional (zero‐dimensional (0D), 1D and 2D) materials to overcome these obstacles. Despite notable advancements, significant challenges remain, such as dark current issues, inefficient photon capture, and suboptimal charge separation in low‐dimensional materials. For example, 0D materials like PbS quantum dots^[^ ^11^ ^]^ are hindered by poor light absorption and synthesis, impacting their detectivity. 1D materials, such as ZnO nanowires, offer high carrier mobility but face challenges such as low quantum efficiency and integration difficulties with larger semiconductor processes.^[^ ^12^ ^]^ Similarly, 2D counterparts, including graphene, black phosphorus (bP), and transition metal dichalcogenides, hold potential for infrared photodetection but are hampered by issues like high dark current, susceptibility to atmospheric oxidation, and difficulties in achieving broad photoresponse and high responsivity.^[^ ^13^, ^14^, ^15^, ^16^, ^17^, ^18^, ^19^ ^]^ Addressing the specific limitations of each material class remains a critical endeavor for advancing infrared photodetection technology.

The unique advantages of 2D layered materials, characterized by the absence of dangling bonds, enable the construction of van der Waals (vdWs) heterostructures that offer enhanced properties beyond those of the individual constituent materials alone.^[^ ^20^, ^21^, ^22^, ^23^, ^24^ ^]^ Consequently, various heterogeneous constructions with built‐in electric fields have been engineered to reduce the large dark current and enhance the photovoltaic performance, as exemplified by the Schottky junction barrier‐band‐aligned graphene/MoTe_2_/graphene vdW heterostructure,^[^ ^25^ ^]^ achieving a 0.11 A·W^−1^ responsivity at 1064 nm through the gate voltage modulation of two Schottky barriers, the WS_2_/GaAs p–n junction,^[^ ^26^ ^]^ which features Zener tunneling with an ultra‐high switching ratio of 10^7^ across a broadband spectral response from 200 to 1550 nm, and the WSe_2_ lateral p–i–n homojunction,^[^ ^27^ ^]^ leveraging slow photoresponse dynamics to achieve an ultra‐fast 264 ns response time. Simultaneously, the bP/MoS_2_ heterostructure achieves an external quantum efficiency of 35% at room temperature in the mid‐wave infrared (MWIR) region, with a specific detection rate as high as 1.1 × 10^10^ cm·Hz^1/2^·W^−1.[^ ^28^ ^]^ Meanwhile, integrating graphene with alternating bands of WS_2_/WSe_2_ enables a tradeoff between a high response rate of 1.7 × 10^7^ mA·W^−1^ and a fast response time of 3–4 µs by utilizing alternating electron and hole conduction channels.^[^ ^29^ ^]^ Therefore, the meticulous design of band alignment in heterostructures based on 2D materials is imperative for optimizing device performance. Van der Waals heterojunctions can be designed to optimize absorption for specific infrared bands, and the detectivity and responsivity may still be limited by the absorption efficiency and carrier transport characteristics of the material itself. Addressing the alignment of energy bands not only facilitates improved carrier dynamics but also plays a pivotal role in overcoming the issues related to limited responsivity and the narrow optical response spectra, thereby maximizing the device's utility in photodetection. Topological insulators (TIs), representing a novel phase of quantum matter, exhibit a sizable gap in the bulk and spin‐momentum‐locked Dirac cones on their surfaces.^[^ ^30^, ^31^ ^]^ With Dirac‐like surface states as the topological characteristic of bulk wavefunction, TI emerges as an intriguing material platform for the exploration of high‐performance broadband photodetection across the infrared to terahertz range.^[^ ^32^ ^]^ Furthermore, artificial created vdWs heterostructures, featuring a type‐II staggered energy band alignment, can facilitate interlayer excitation and enable the leapfrogging of sub‐bandgap photons, thereby promoting self‐powered phenomena and rendering it well‐suited for portable and wearable applications.

Herein, a type‐II Ta_2_NiSe_5_/Bi_2_Se_3_ vdWs heterojunction photodetector device has been constructed by making use of the ideal bandgap and excellent optoelectronic properties of Ta_2_NiSe_5_ and Bi_2_Se_3_ nanoflakes. In particular, the type‐II band alignment between 2D semiconductors facilitates interlayer excitation and transition of sub‐bandgap photons at telecom wavelengths. The strategic construction of heterojunctions through the energy band alignment of Ta_2_NiSe_5_ and Bi_2_Se_3_ contributes to the development of a cost‐effective high‐performance photodetector. This device achieves a peak detectivity of 3.8 × 10^11^ cm·Hz^1/2^·W^−1^ under 1064 nm laser irradiation, with a responsivity exceeding 0.48 A·W^−1^. Demonstrating an ultra‐broadband photoresponse from the visible to the MWIR spectrum (520 nm–4.65 µm) at room temperature, it underscores its vast potential in broadband light detection. Furthermore, employing Ta_2_NiSe_5_/Bi_2_Se_3_ heterojunction photodetectors as signal receivers has led to the development of novel information transmission processes and imaging techniques, showcasing its potential in infrared communication and imaging systems.

## 2 Results and Discussion

**Figure 1:**
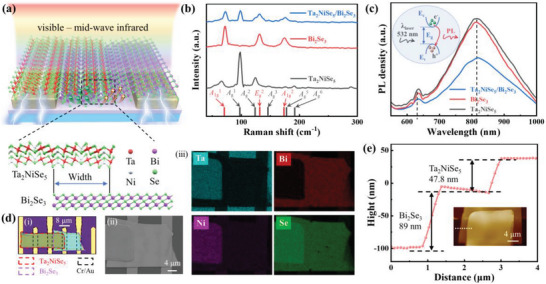
Characterizations of the Ta_2_NiSe_5_/Bi_2_Se_3_ heterostructure on the SiO_2_/Si substrate. a) A structural diagram of the Ta_2_NiSe_5_/Bi_2_Se_3_ vdWs heterojunction photodetector and atomic structure of Ta_2_NiSe_5_ and Bi_2_Se_3_. b) Raman plots corresponding to Ta_2_NiSe_5_, Bi_2_Se_3_ flakes and the heterojunction region. The lower layers are labeled according to their Raman peaks. c) PL spectra corresponding to Ta_2_NiSe_5_, Bi_2_Se_3_ flakes and the heterojunction region. The laser excitation wavelength is 532 nm and the embedded figure shows a schematic diagram of the PL quenching process. d) i) Optical microscopy image of the Ta_2_NiSe_5_/Bi_2_Se_3_ vdWs heterojunction (The gold inter‐electrode channel is 8 um); ii) SEM electron microscopy (The scale bar is 4 µm) and iii) EDS spectra of the photodetector. e) AFM morphology of the heterojunction region and its corresponding thickness measured along the white dashed line (The scale bar is 4 µm).

Engineering heterostructures of 2D materials provides a pivotal strategy to enhance light‐matter interactions and thereby advances the development of high‐performance, energy‐efficient, and compact photodetectors. Conventional 2D material‐based photodetectors have been constrained by the necessity of high bias voltages for efficient photocarriers collection, primarily due to inherently high resistance and the presence of Schottky barriers. A band‐alignment‐based device using the Ta_2_NiSe_5_/Bi_2_Se_3_ heterostructure is tailored specifically for infrared detection and applications, as seen in **Figure** **1**a. The detailed atomic structure of the Ta_2_NiSe_5_ and Bi_2_Se_3_ flakes, featuring an 8 µm wide overlap region, is illustrated in the enlarged view. By employing a dry transfer technique, the overlapping regions of the Ta_2_NiSe_5_/Bi_2_Se_3_ heterojunction are intentionally positioned at the center of the metal electrodes, effectively minimizing the lateral transport distance of photogenerated carriers and mitigating the impact of series resistance. The assembly of the Ta_2_NiSe_5_ and Bi_2_Se_3_ heterostructure is confirmed through Raman spectroscopy, as shown in Figure 1b. The Raman spectrum exhibits characteristic peaks of both Ta_2_NiSe_5_ and Bi_2_Se_3_, with a notable weakening of these peaks in the overlap region (indicated by the black dashed line), which indicates that the heterojunction has a high interfacial quality with strong interlayer coupling effects.^[^ ^33^ ^]^ The identified peaks at 98.2, 123.8, 146.6, 176, and 190.7 cm^−1^ represent the in‐plane vibrational mode (*A*_g_^1^, *A*_g_^2^, *A*_g_^3^, *A*_g_^5^, *A*_g_^6^) of Ta_2_NiSe_5_, respectively, aligning with previous literature.^[^ ^34^, ^35^ ^]^ Additionally, the peaks at 72.45 cm^−1^ (out‐of‐plane vibration mode *A*_1g_^1^), 131.8 cm^−1^ (in‐plane vibration mode *E*_g_^2^), and 174.7 cm^−1^ (out‐of‐plane vibration mode *A*_1g_^2^) are attributed to Bi_2_Se_3_, as reported by others.^[^ ^36^ ^]^ To experimentally ascertain the interlayer charge transfer dynamics between Ta_2_NiSe_5_ and Bi_2_Se_3_, room‐temperature Photoluminescence (PL) spectra were acquired for both individual flakes and the heterojunction, as presented in Figure 1c. The schematic diagram of the optical measurement system is shown in Figure S1 (Supporting Information). As can be seen in the inset of Figure 1c, with the excitation of the 532 nm laser, the electrons at the top of the valence band are stimulated to jump to the excited electronic state, and then return to the low‐energy state after a relaxation process in terms of energy and momentum (which releases the photons). The PL spectra reveals that the Ta_2_NiSe_5_ and Bi_2_Se_3_ flakes exhibit prominent emission peaks at 818 nm and a weaker peak at 638 nm, corresponding to the A exciton peak and indirect transition I peak, respectively.^[^ ^37^, ^38^ ^]^ Compared with single Ta_2_NiSe_5_ or Bi_2_Se_3_, the PL intensities of the heterojunction region drops dramatically at 638 and 818 nm, indicating the effective carriers generation and separation across the heterojunction, leading to the increase of non‐radiative recombination and the decrease of PL emission recombination. The morphological integrity of the Ta_2_NiSe_5_/Bi_2_Se_3_ heterojunction is further corroborated by a Scanning Electron Microscope (SEM) image, as shown in Figure 1d‐ii, revealing a smooth and uniform interface. Meanwhile, the element distribution and composition analysis are further investigated by an energy dispersive spectrometer (EDS), as shown in Figure 1d‐iii, which confirms the homogeneous spatial distributions of Ta, Ni, and Bi, with the atomic molar ratio aligning with the stoichiometric composition of the Ta_2_NiSe_5_/Bi_2_Se_3_ heterojunction (Figure S2, Supporting Information). Atomic Force Microscopy (AFM) measurements, presented in Figure 1e, determined the thicknesses of the Ta_2_NiSe_5_ and Bi_2_Se_3_ films to be ≈47.8 and 89 nm, respectively.

**Figure 2:**
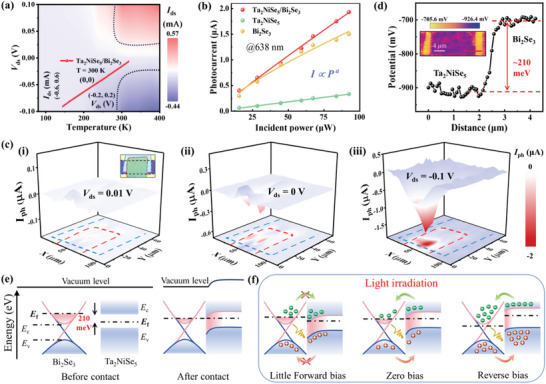
Energy band and electrical characteristics of the Ta_2_NiSe_5_/Bi_2_Se_3_ heterostructure. a) Contour plots of the current characteristics of the device at various temperatures and bias voltages. The inset image is the I_ds_−V_ds_ curve of the Ta_2_NiSe_5_/Bi_2_Se_3_ heterojunction photodetector in darkness at room temperature. b) The fitting photocurrent versus laser incident power of Ta_2_NiSe_5_, Bi_2_Se_3_ flakes, and heterojunction at 638 nm under 0.1 V bias by the power law. c) Photocurrent mapping images correspondingly measured at 0.01, 0, and −0.1 V bias under 638 nm laser illumination (The scale bar is 4 µm). The inset image is the optical microscope image corresponding to the scanned area of the device. d) The contact potential difference curve between Ta_2_NiSe_5_ and Bi_2_Se_3_ along the red line. The inset image is the KPFM image of the Ta_2_NiSe_5_/Bi_2_Se_3_ heterojunction photodetector (The scale bar is 4 µm). e) Schematic band alignments between Bi_2_Se_3_ and Ta_2_NiSe_5_ before and after contact. E_c_ (Bi_2_Se_3_) = −4.91 eV, E_v_ (Bi_2_Se_3_) = −5.21 eV, E_c_ (Ta_2_NiSe_5_) = −4.6 eV, E_v_ (Ta_2_NiSe_5_) = −4.93 eV. f) Energy band alignment diagrams of Bi_2_Se_3_/Ta_2_NiSe_5_ heterojunction at forward, zero and reverse bias voltages under light irradiation conditions, respectively.

Figure 1a delineates a schematic representation of a photodetector predicated on a type‐II Ta_2_NiSe_5_/Bi_2_Se_3_ vdWs heterojunction with its corresponding optical image elucidated in Figure 1d. To assess the influence of the thermal effect on the Ta_2_NiSe_5_/Bi_2_Se_3_ heterojunction band alignment, we analyzed the *I*_ds_ in relation to *V*_ds_ and temperature under dark conditions (78‐400 K), presenting the results in **Figure** **2**a. The inset of Figure 2a and Figure S3c (Supporting Information) demonstrate that both the Ta_2_NiSe_5_ and Bi_2_Se_3_ flakes, as well as the heterojunction photodetector, establish good Ohmic contact with the Cr/Au electrodes, evidenced by the linearly increasing current with *V*_ds_, thereby negating any significant influence from electrode contacts. The thermionic emission model, employed in Figure S3b (Supporting Information) and the applied to data extracted from Figure 2a at minimal forward bias, shows an approximation of the linear dependency, affirming the suitability of the model for the photodetector.^[^ ^39^ ^]^ Figure 2b depicts a fitting of the incident power‐dependent photocurrent to a power law (*I*_ph_∼*AP* ^α^), where *I*_ph_ is the photocurrent, and α determines the photoresponse to the incident power, giving the power exponents of 0.996, 0.993, and 0.975 for the Ta_2_NiSe_5_/Bi_2_Se_3_ heterojunction, Ta_2_NiSe_5_, and Bi_2_Se_3_ flakes, respectively. This data indicates a more pronounced linear relationship between photocurrent and incident light power in the heterojunction compared to the individual materials. The current‐voltage curves for various incident optical powers at 638 nm, as shown in Figure S4a–c (Supporting Information), further corroborate this finding. Figure S4d (Supporting Information), depicting the photoresponse as a function of incident power and *V*_ds_, reinforces the power dependency of the photodetector. To investigate the photoresponse mechanism of the photodetector in the visible/NIR wavelength, the photocurrent mapping is measured with micro‐area laser scanning. Figure 2c and Figure S5 (Supporting Information) show photocurrent mappings under infrared illumination with/without bias voltages, respectively, where the heterojunction area is marked with blue and red dashed boxes, and the corresponding scanning optical microscope image is shown in the inset. The mappings reveal a pronounced photoresponse primarily in the effective overlap area between Ta_2_NiSe_5_ and Bi_2_Se_3_.

Next, Kelvin probe force microscopy (KPFM) was employed to elucidate the surface potential distribution and the built‐in field at the heterojunction interface of the Bi_2_Se_3_/Ta_2_NiSe_5_ system. The 2D KPFM mapping, as depicted in the inset of Figure 2d, reveals the surface potential variations between the AFM tip and the heterojunction. The contact potential difference between the Bi_2_Se_3_ and Ta_2_NiSe_5_ flakes is ≈210 meV, with a lateral depletion spacing of ≈1 µm, a spacing that notably exceeds the flake thickness, thereby substantiating the presence of a significant built‐in potential barrier at the Bi_2_Se_3_/Ta_2_NiSe_5_ interface. Work functions for Bi_2_Se_3_ (4.6 eV) and Ta_2_NiSe_5_ (4.81 eV) were deduced from these KPFM measurements, detailed in Figure S7 (Supporting Information). Based on the above‐measured data and previous reports,^[^ ^40^, ^41^, ^42^, ^43^, ^44^, ^45^ ^]^ we present a schematic representation of the energy band alignment for Bi_2_Se_3_ and Ta_2_NiSe_5_, pre and post‐contact, in Figure 2e. This illustration depicts a type‐II band alignment, characterized by a Fermi level difference (Δ) of 0.21 eV, defined by Δ = *Φ*_Ta2NiSe5_ − *Φ*_Bi2Se3_. Post‐contact, the energy levels near the Ta_2_NiSe_5_ interface exhibit an upward bend, forming a built‐in potential region. This configuration minimizes electron transfer to Bi_2_Se_3_, culminating in an electron accumulation in Bi_2_Se_3_ and hole accumulation in Ta_2_NiSe_5_, until thermal equilibrium is attained. Complementing these observations, Raman spectroscopy and the marked diminution in PL intensity at the Ta_2_NiSe_5_/Bi_2_Se_3_ heterostructure corroborate the charge transfer phenomenon within the heterostructure. The far‐right segment of Figure 2f illustrates the photodetection mechanism: upon illumination, photoexcited electrons in Ta_2_NiSe_5_ traverse the barrier layer to the n‐type Bi_2_Se_3_ layer, serving as the majority carriers. This transfer results in a notable PL quenching in Ta_2_NiSe_5_. Consequently, the recombination of photogenerated electron–hole pairs in Ta_2_NiSe_5_ is suppressed, enhancing the absorption efficiency and facilitating a substantial photogenerated current.

**Figure 3:**
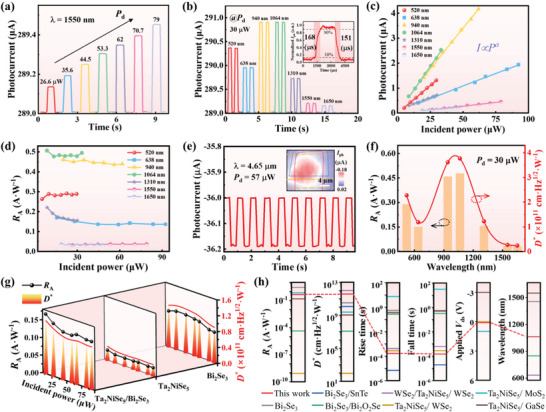
Photoresponse characteristics of the Ta_2_NiSe_5_/Bi_2_Se_3_ heterostructure. a) Optical switching characteristics measured at 1550 nm laser at different incident powers at V_ds_ = 0.1 V. b) Real‐time photoresponse properties of the device under different‐wavelength (520–1650 nm) light illumination at 0.1 V bias and 30 µW fixed incident power. The inset is the response time of the heterojunction photodetector at 1064 nm. Rise time of 168 µs (fall time of 151 µs) is measured from 10–90% (from 90‐10%) of the maximum photocurrent after light is switched on (off). c) The fitting photocurrent versus laser incident power of the device under different‐wavelength light illumination at 0.1 V bias by the power law. d) R_A_ of the device dependent on laser incident power at 0.1 V bias voltage under different‐wavelength light illumination. e) Photoswitching response of the heterojunction photodetector under 4.65 µm laser illumination with P_d_ = 57 µW at zero bias (The modulation frequency is 1 Hz). The inset is photocurrent mapping images correspondingly measured at zero bias under 4.65 µm laser illumination (The scale bar is 4 µm). f) R_A_ and D^*^ under different wavelengths of 520–1650 nm at 0.1 V bias and 30 µW fixed incident power. g) Incident power depended on R_A_ and D^*^ of Ta_2_NiSe_5_, Bi_2_Se_3_ flakes, and heterojunction at 638 nm under 0.1 V bias. h) The comparison of performance parameters for photoelectric detection between the Ta_2_NiSe_5_/Bi_2_Se_3_ heterostructure photodetector and other high‐performance photodetectors based on 2D materials, including Bi_2_Se_3_,^[^ ^36^ ^]^ Bi_2_Se_3_/SnTe,^[^ ^55^ ^]^ Bi_2_Se_3_/Bi_2_O_2_Se,^[^ ^56^ ^]^ WSe_2_/Ta_2_NiSe_5_/WSe_2_,^[^ ^57^ ^]^ Ta_2_NiSe_5_/WSe_2_,^[^ ^58^ ^]^ Ta_2_NiSe_5_/MoS_2_,^[^ ^45^ ^]^ Ta_2_NiSe_5_/GaSe.^[^ ^59^ ^]^

During our investigation on the broadband photoresponse capabilities of the Ta_2_NiSe_5_/Bi_2_Se_3_ heterojunction photodetector, distinct photocurrent switching characteristics under varying laser powers were observed. As depicted in **Figure** **3**a, the photoresponse at 1550 nm shows a clear correlation between increasing laser power and photocurrent enhancement. This behavior is indicative of an augmented generation of photogenerated carriers proportional to the absorbed incident photons. Further exploration of the real‐time photoresponse properties under diverse wavelength illuminations (520‐1650 nm), including those within the optical communication band (830, 1310, and 1550 nm), is presented in Figure 3b. Notably, the heterojunction photodetector exhibits pronounced wavelength selectivity, particularly at 940 and 1064 nm, and maintains stable photoresponse states, corroborating our previous PL spectra findings. As shown in the inset of Figure 3b, the response time at 1064 nm is relatively fast. A systematic analysis of the photoresponse as a function of optical power across different wavelength bands is provided in Figure S8 (Supporting Information), revealing a consistent and swift photoresponse in both the visible and near‐infrared spectral regions. A linear laser power‐dependent photocurrent behavior (*I*_ph_∼*AP* ^α^) is observed in Figure 3c, in which the values of α are 0.998, 0.997, 0.998, 0.992, 0.99, 0.992, and 0.985, respectively, attributed to the intricate interplay of electron–hole pair generation, recombination, and trapping dynamics within the heterojunction.^[^ ^46^, ^47^ ^]^ The performance of the photodetector is rigorously quantified through a comprehensive assessment of response time, external quantum efficiency (EQE), noise‐equivalent power (NEP), responsivity (*R*_A_), and specific detectivity (*D* ^*^) with the following equations:^[^ ^48^ ^]^ *R*_A_ = *I*_ph_/*P*_eff_ = (*I*_ds_‐*I*_d_)/(*P*_in_·*A*_eff_), where *I*_ph_ is the photocurrent, *I*_d_ is the dark current, *P*_eff_ is the effective incident‐light intensity, *P*_in_ is the power of incident light, and *A*_eff_ is the effective illuminated area, respectively. *D* ^*^ represents the ability of a photodetector to detect weak optical signals, as expressed by the equation *D* ^*^ = (A·∆f/NEP)^−1/2^ = ((A·∆f)^−1/2^·*R*_A_)/*i*_n_, where A is the effective area of the device, Δf = 1 Hz and the noise‐equivalent power can be expressed as NEP = *i*_n_/*R*_A_. Typically, the total noise current (*i*_n_) of the photodetectors includes thermal noise (*i*_t_), shot noise (*i*_s_), g‐r noise, and 1/f noise.^[^ ^49^ ^]^ Figure S11a (Supporting Information) displays the test results for the noise power spectrum of the device. As high frequencies are utilized for sampling in the performance test, 1/f noise can be disregarded. Therefore, we calculate the total noise current from the thermal noise and the shot noise: *i*_n_^2^ = *i*_t_^2^+*i*_s_^2^ = (2q*i*_d_+(4*k*_B_·T)/R)·∆f, where q is the elementary charge, *i*_d_ is the dark current, *k*_B_ is the Boltzmann constant, T is the thermodynamic temperature, R is the resistance and Δf is the bandwidth. Based on the above equation, the evaluation of the linear dynamic‐stability is based on the variation of the responsivity, as shown in Figure 3d. The responsivity curve, predominantly linear with minor fluctuations, validates the excellent linear dynamic stability of the photodetector.

Traditionally, the limitations of mid‐wavelength infrared photodetectors necessitate stringent external conditions, such as cooling and bias.^[^ ^10^ ^]^ Here we explore the self‐powered photoresponse of the device at room temperature under 4.65 µm infrared radiation in Figure 3e. A notable reduction in photocurrent and responsivity, as compared to the photoresponse at 638 and 1550 nm (Figure S10a,b, Supporting Information),^[^ ^29^, ^50^, ^51^, ^52^ ^]^ suggests a distinct photoresponse mechanism in the mid‐wavelength infrared spectrum. Unlike the interband transition of charges governing the visible to near‐infrared range, the mid‐wavelength infrared mechanism predominantly relies on the contribution of topological surface state due to the photon energy being much lower than the bulk bandgap of Bi_2_Se_3_.^[^ ^32^ ^]^ The existence of a gapless Dirac surface states, protected by time‐reversal symmetry, is excited by the mid‐infrared photons. The electron–hole pairs are separated under the action of the built‐in electric field from the heterojunction structure and are ultimately collected by the metal electrodes, producing self‐powered photocurrent.^[^ ^1^, ^53^, ^54^ ^]^ The photocurrent mapping at 4.65 µm as shown in the inset of Figure 3e, reveals a stable photoresponse concentrated in the overlapped area of the heterojunction, further demonstrating the above explanation. The extended optical response time under mid‐wavelength infrared irradiation, relative to visible and near‐infrared, is ascribed to the lower energy of mid‐wavelength infrared photons, which impedes rapid saturation of photogenerated carriers.^[^ ^19^ ^]^ Figure 3f and Figure S11b (Supporting Information) illustrate the changes in the sensitivity characterization parameters of the heterojunction photodetector as the response wavelength varies. EQE, defined as EQE = *h*c*R*_A_/eλ (where *h* is the Planck constant and c is the speed of light), reflects the ratio of photoexcited carriers to incident photons. With the dark current of 2.89 × 10^−4^ A at 0.1 V, the thermal noise and shot noise are estimated to be 9.62 and 7.02 pA·Hz^−1/2^, respectively, within a bandwidth of 1 Hz. The heterojunction photodetector achieves a peak photosensitivity at 1064 nm, yielding a photoresponsivity of 0.48 A·W^−1^ and a specific detectivity of 3.8 × 10^11^ cm·Hz^1/2^·W^−1^. Responsivity and detectivity for the 1550 nm laser reach 0.03 A·W^−1^ and 0.27 × 10^11^ cm·Hz^1/2^·W^−1^, respectively, adequate for subsequent optical communication and imaging applications. Figure 3g showcases the incident power dependence of responsivity and detectivity for the Ta_2_NiSe_5_/Bi_2_Se_3_ heterojunction, as well as individual Ta_2_NiSe_5_ and Bi_2_Se_3_, under 638 nm visible light irradiation. The heterojunction exhibits maximum *R*_A_ and *D* ^*^ values of ≈0.17 A·W^−1^ and 1.34 × 10^11^ cm·Hz^1/2^·W^−1^, respectively, which are higher than those of the Ta_2_NiSe_5_ (∼0.03 A·W^−1^, ∼0.35 × 10^11^ cm·Hz^1/2^·W^−1^) and Bi_2_Se_3_ (∼0.11 A·W^−1^, ∼1.11 × 10^11^ cm·Hz^1/2^·W^−1^) photodetectors. This enhanced detection of minimal illumination signals is attributed to the type‐II heterojunction's optimal energy band alignment. Figure 3h and Table S1 (Supporting Information) summarize the performance of the main 2D material‐based photodetectors with their highest records. A comparison of the *R*_A_, *D* ^*^, response time and detection wavelength found for the aforementioned 2D photodetectors and our fabricated heterostructure photodetector demonstrates that our device exhibits excellent comprehensive performance. The optoelectronic heterostructure exhibits excellent performance, making it a promising choice for various applications such as image sensing, communications, environmental monitoring, and remote control.

**Figure 4:**
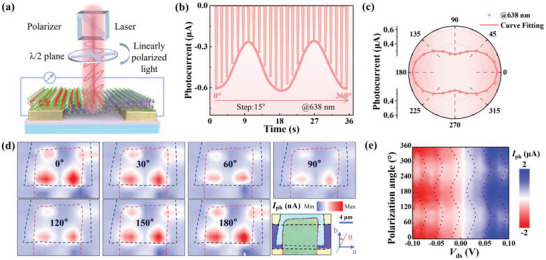
Polarization‐resolved Ta_2_NiSe_5_/Bi_2_Se_3_ heterojunction photodetector. a) Schematic diagram of polarization photoresponse measurement. b) Polarized angle‐dependent photocurrent at zero bias voltage under 638 nm laser. c) Experimental normalized angle‐resolved photocurrents are plotted with the linear‐polarization laser of 638 nm at zero bias voltage in the polar coordinates. The corresponding lines are the fitting results using the sinusoidal function. d) Polarized photocurrent mapping using 638 nm laser irradiation at zero bias voltage with a polarization angle of 0° to 180°. The lower right corner is the optical microscope image corresponding to the scanned region of the device (The scale bar is 4 µm). e) Contour map of polarized photoelectric properties of the device for different polarization angles under 638 nm light irradiation and different bias voltages.

In this study, we explore the polarization‐resolved photodetection capabilities of Ta_2_NiSe_5_/Bi_2_Se_3_ heterojunctions, leveraging their anisotropic optical properties to add a new dimension to infrared detection. **Figure** **4**a presents a schematic illustrating the detection of polarization‐sensitive characteristics by our detector. Linearly polarized light, achieved through a polarizer, is further modulated by a half‐wave plate to adjust the polarization angle. We define the direction parallel to the gold electrode as the “a‐axis”, and the initial incidence angle of the linearly polarized light as the “b‐axis”, perpendicular to the “a‐axis”. The polarization angle θ is then the angle between the linearly polarized light and the “b‐axis”. Our polarization performance tests, conducted under 638 nm laser irradiation without external bias, are detailed in Figure 4b. Here, the photocurrent exhibits pronounced periodic variations, with maximum and minimum values aligned along the “b‐axis” and “a‐axis” of the heterojunction, respectively. A classic demonstration of the polarization‐resolved performance of the Ta_2_NiSe_5_/Bi_2_Se_3_ heterojunction photodetector is shown in Figure 4c, which was measured with the illumination of a linearly polarized laser at 638 nm. The experimental data points and the corresponding fitting curve, modeled by the sine function^[^ ^60^ ^]^ *I*_ph_ (θ) = *I*_py_·cos^2^ (θ+φ) + *I*_px_·sin^2^ (θ+φ), are shown, where *I*_ph_ is the photocurrent of the photodetector; θ is the polarization angle; and *I*_px_, *I*_py_, and φ are the fitting parameters. The polarization ratio defined by *I*_ph max_/*I*_ph min_ is ≈2.83 with 638 nm laser illumination. To comprehensively confirm the polarization sensitivity of the photodetector, we applied illumination of a linearly polarized laser, which is at 520, 940, 1064, 1310, 1550, and 1650 nm. The device exhibited distinct polarization‐resolved characteristics under all tested conditions (for details, refer to Figure S12, Supporting Information). At 520 nm, the polarization ratio is significantly high at 3.42. This could be attributed to surface and interface effects dominating the polarization interaction at shorter wavelengths. The surface of Ta_2_NiSe_5_ may exhibit enhanced sensitivity to specific polarization states, particularly when special electronic states formed at the interface with Bi_2_Se_3_ exhibit pronounced polarization‐selective absorption at this range. From 940 to 1650 nm (near‐infrared region), the polarization ratio reduced from 2.28 to 1.27. The reduced polarization dependency at these wavelengths may reflect a decrease in polarization‐dependent electronic transitions within the Ta_2_NiSe_5_ and Bi_2_Se_3_. The difference could be related to multiple interface effects and intrinsic absorption characteristics of the materials at these specific wavelengths. These parameters are higher compared to those of Ta_2_NiSe_5_, Bi_2_Se_3_ photodetectors, and other anisotropic photodetectors (shown in Table S1, Supporting Information). The Ta_2_NiSe_5_/Bi_2_Se_3_ heterojunction photodetector thus emerges as a promising candidate for polarization‐sensitive optical sensing, eliminating the need for additional optical modules. The origin of the detector's polarization detection capability is further corroborated by polarized photocurrent mapping. As depicted in Figure 4d, the photocurrent mapping across various polarization angles (ranging from 0° to 180°) reveals a clear maximum at 0° and a significant minimum at 90° in the overlapping region between Ta_2_NiSe_5_ and Bi_2_Se_3_. The 2D mapping of polarized photocurrent shown in Figure 4e demonstrates dependence on the polarization angle and *V*_ds_ clearly. It is evident that the measured photocurrent is dependent on the bias voltage and the polarization angle.

**Figure 5:**
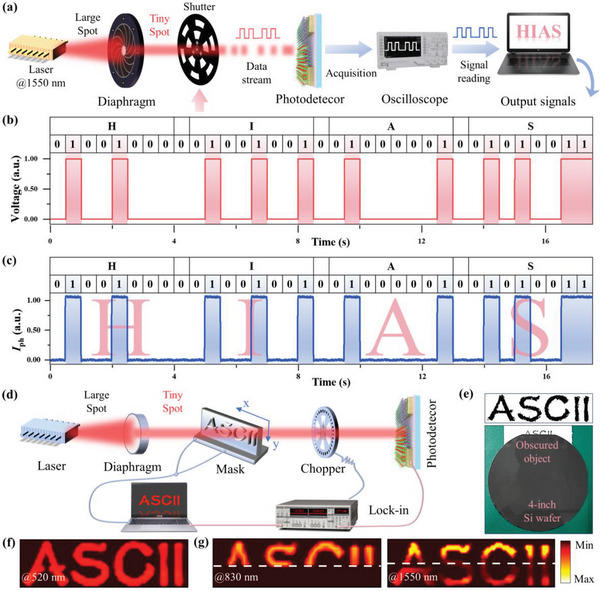
The utilization of near‐infrared communication and imaging. a) Schematic illustration of the infrared communication system employing the Ta_2_NiSe_5_/Bi_2_Se_3_ heterojunction photodetector as a signal receiver. b) The input signals that control the mechanical shutter to modulate the laser. c) The photocurrent received by the Ta_2_NiSe_5_/Bi_2_Se_3_ heterojunction photodetector is modulated by the light intensity in b) and the “HIAS” is decoded. d) Schematic illustration of the imaging system in the visible and near‐infrared wavelength bands. e) Photographs of the complete “ASCII” object and the “ASCII” object obscured by a 4‐inch silicon wafer. The imaging scanning area is 3 cm × 1 cm. f) The high‐resolution imaging result of the complete “ASCII” object under 520 nm. g) Imaging pattern of the “ASCII” object obscured by a 4‐inch silicon wafer under 830 and 1550 nm.

In addition, we have capitalized on the rapid response capabilities of our room temperature infrared detector, based on the Ta_2_NiSe_5_/Bi_2_Se_3_ heterostructure, to pioneer its application in infrared (IR) optical communication. Notably, the operational windows for optical communication, including the crucial 1550 nm wavelength (the third window), fall within the response spectrum of our device. **Figure** **5**a illustrates the setup for a novel visual demonstration in IR optical communication, utilizing the level signal depicted in Figure 5b to control a shutter mechanism in the communication system. In this configuration, an open shutter allows the light beam to impinge on the photodetector, generating a photocurrent interpreted as a binary “1”. Conversely, a closed shutter blocks the light, resulting in a drop in current, denoted as “0”. The device adeptly converts modulated pulsed IR signals into electrical signals, which are then transmitted to a terminal computer via an oscilloscope. This process successfully produced American Standard Code for Information Interchange (ASCII) codes corresponding to “HIAS”, as shown in Figure 5c, with a “0” inserted as a separator between each letter. The fidelity of the output signal curve to the input signal underscores the device's potential in IR optical communication. Exploiting the sensitivity of the heterojunction device to light intensity and its broad‐band response, we have further extended its application to visible and near‐infrared imaging. Figure 5d shows a schematic of an imaging measurement system in which a single device serves as a sensing pixel, and the laser light passes through a hollow patterned mask fixed on a 2D translation stage and strikes the pixel unit. Subsequently, the position‐dependent photocurrent of the device is recorded by a software‐programmed computer to form a complete high‐quality image. Figure 5e shows the “ASCII” object and its physical image obscured by a 4‐inch silicon wafer, and in Figure 5f, a clear image of the “ASCII” object has been realized with a resolution of up to 0.5 mm under a 520 nm laser. Significantly, the effective optical window of the heterojunction photodetector, spanning 520 to 4650 nm, surpasses the human visual range (≈400–700 nm). This capability positions the device as a compelling component in future clairvoyant optoelectronic vision systems. Fourier Transform Infrared Spectroscopy (FTIR) near‐infrared transmittance tests on the wafer, detailed in Figure S13 (Supporting Information), demonstrate a substantial difference in transmittance under 830 and 1550 nm laser illumination, exceeding a factor of five. This disparity in transmittance rates for visible light and near‐infrared wavelengths is ingeniously harnessed to achieve a dual‐channel imaging effect under shading conditions, as illustrated in Figure 5g. The broad applicability of near‐infrared communication and imaging technologies, as exemplified by our device, holds immense potential across various sectors, including military security, civil navigation, and medical diagnostics. These technologies can significantly enhance data transmission rates and image quality, meeting the growing demands of modern society for efficient communication and precise imaging. Our Ta_2_NiSe_5_/Bi_2_Se_3_ heterojunction photodetector, with its rapid response, broad spectral sensitivity, and high‐resolution imaging capabilities, represents a substantial leap forward in the field of infrared detection and its practical applications in optical communication and beyond.

## 3 Conclusion

In summary, our work has successfully demonstrated the design and fabrication of a type‐II Ta_2_NiSe_5_/Bi_2_Se_3_ heterojunction photodetector, which operates at room temperature and is tailored for imaging and optical communication applications. The heterojunction demonstrates superior performance (1.34 × 10^11^ cm·Hz^1/2^·W^−1^) compared to its constituent materials (0.35 × 10^11^ cm·Hz^1/2^·W^−1^ for Ta_2_NiSe_5_ and 1.11 × 10^11^ cm·Hz^1/2^·W^−1^ for Bi_2_Se_3_). Through meticulous PL, KPFM analysis, and photocurrent testing, we have deciphered the photoresponse mechanism of the photodetector, which incorporates a photovoltaic effect within the heterojunction region under 520 nm‐4.65 µm laser irradiation. The device exhibits a competitive *R*_A_ of 0.48 A·W^−1^, a low NEP of 0.249 × 10^−10^ W·Hz^−1/2^, and a high *D* ^*^ of 3.8 × 10^11^ cm·Hz^1/2^·W^−1^, coupled with a fast photoresponse speed (*τ*_r_ ≈168 µs, *τ*_f_ ≈151 µs) under 1064 nm laser illumination. These achievements underscore the potential of finely designed band alignment and device structures in enhancing the capabilities of infrared detectors. Such advancements are pivotal for improving data transmission rates and image quality, thereby fulfilling the demands of modern society for efficient communication and precise imaging. This work not only contributes to the fundamental understanding of heterostructure‐based photodetectors but also opens new avenues for their application in diverse fields, ranging from secure communication to high‐resolution imaging.

## 4 Experimental Section

### Sample and Device Preparation

The bulk Ta_2_NiSe_5_ and Bi_2_Se_3_ were purchased from the Shanghai Onway Technology Co., Ltd. The 2D Ta_2_NiSe_5_ and Bi_2_Se_3_ nanoflakes were obtained by using the mechanical stripping method to strip them from the bulk crystal. The 2D Bi_2_Se_3_ nanoflake was first transferred onto a low resistance silicon substrate with a 300 nm insulating layer of SiO_2_ via poly(dimethylsiloxane) (PDMS), and then the 2D Ta_2_NiSe_5_ nanoflake was stacked onto the Bi_2_Se_3_. The bottom electrodes were patterned using Semi‐Automated Mask Aligner (MA/BA6 Gen4) and then metal contacts (Cr/Au = 5 nm/50 nm) were deposited by high vacuum evaporation technology (Ei‐5Z), and then they went through a lift‐off process.

### Material Characterization

The heterojunction was analyzed using SEM and EDS spectrum analyzer (Gemini500) to determine its surface topography and elemental composition. The thickness of the Ta_2_NiSe_5_ and Bi_2_Se_3_ nanoflakes was performed using an AFM (Dimension ICON). The micro‐Raman system (Renishaw inVia) with a 532 nm laser was performed to obtain the Raman spectrum. The PL spectra was characterized by the confocal microscope (Horiba Scientific LabRAM HR Evolution) and a sequential wavelength 532 nm laser.

### Electronic Properties and Polarization Photocurrent Characterization

Both electrical and optoelectronic properties were characterized in ambient air at room temperature. A highly sensitive dual‐channel digital source meter (Keithley 6482) was used for applying the bias and measuring the current and photovoltage at the same time. The Ta_2_NiSe_5_/Bi_2_Se_3_ heterojunction photodetector was mounted on a sophisticated sample holder to increase stability and precision during measurement. For the basic photoelectric test and performance calculation, the incident light path of the laser was a uniform spot with a spot diameter of 27 µm (520–1650 nm) and 3 µm (4.65 µm), focused by using 50X objective lens. The effective light power density (*P*_eff_) was calculated using *P*_eff_ = *P*_in_ × *A*_eff_ = *P*_in_ × *A*_device_/*A*_spot_, where the effective photoresponse area of the heterojunction *A*_device_ = 8 µm × 11 µm = 88 µm^2^. The photocurrent mapping was performed by scanning the pressure point console in horizontal and vertical directions under the irradiation of a focused modulated laser, and the photocurrent signal was obtained using a lock‐in amplifier (MStarter 200). Linearly polarized light was obtained by adding the Glan‐Taylor prism and half‐wave plate on the path of the laser, and the photoelectronic measurements were measured by rotating the half‐wave plate. Linearly polarized light was measured by adding a Glan‐Taylor Prism and a half‐wave plate in the path of the laser and rotating the half‐wave plate to measure the optoelectronic measurements.

## Conflict of Interest

The authors declare no conflict of interest.

## Supporting information

Supporting Information

## Data Availability

The data that support the findings of this study are available in the supplementary material of this article.
