# Supplementary material for: Room‐Temperature Band‐Aligned Infrared Heterostructures for Integrable Sensing and Communication
Source: Adv Sci (Weinh). 2024 Jun 5;11(36):2401716. doi: 10.1002/advs.202401716 (PMC11423140; doi:10.1002/advs.202401716)
Supplement: Supplementary file 1 — Supporting Information [file ADVS-11-2401716-s001.pdf]

ADVANCED SCIENCE
Open Access

Supporting Information

for *Adv. Sci.*, DOI 10.1002/advs.202401716

Room-Temperature Band-Aligned Infrared Heterostructures for Integrable Sensing and Communication

Kening Xiao, Shi Zhang, Kaixuan Zhang, Libo Zhang, Yuanfeng Wen, Shijian Tian, Yunlong Xiao, Chaofan Shi, Shicong Hou, Changlong Liu*, Li Han, Jiale He, Weiwei Tang, Guanhai Li, Lin Wang* and Xiaoshuang Chen**

Supporting Information

Room-Temperature Band-aligned Infrared Heterostructures for Integrable Sensing and Communication

Kening Xiao[1, 2†], *Shi Zhang*[1, 2†], *Kaixuan Zhang*[1, 2†], *Libo Zhang*[1, 2*], *Yuanfeng Wen*[1], *Shijian Tian*[2], *Yunlong Xiao*[1], *Chaofan Shi*[1], *Shicong Hou*[1, 4], *Changlong Liu*[1*], *Li Han*[1, 2], *Jiale He*[1, 2], *Weiwei Tang*[1], *Guanhai Li*[1, 2], *Lin Wang*[1, 2, 3*] and *Xiaoshuang Chen*[1, 2, 3, 4*]

[1]College of Physics and Optoelectronic Engineering, Hangzhou Institute for Advanced Study, University of Chinese Academy of Sciences, No. 1, Sub-Lane Xiangshan, Xihu District, Hangzhou 310024, China.

[2]State Key Laboratory of Infrared Physics, Shanghai Institute of Technical Physics, Chinese Academy of Sciences, 500 Yu-Tian Road, Shanghai 200083, China.

[3]University of Chinese Academy of Sciences, No. 19A Yuquan Road, Beijing 100049, China.

[4]School of Physical Science and Technology, ShanghaiTech University, Shanghai 201210, China.

* Corresponding authors: Libo Zhang (zhanglibo@ucas.ac.cn); Changlong Liu (clliu@ucas.ac.cn); Lin Wang (wanglin@mail.sitp.ac.cn); Xiaoshuang Chen (xschen@mail.sitp.ac.cn).

Main contents

1. The schematic diagram of the PL optical measurement system

2. The elemental composition analysis of the Ta_2NiSe_5/Bi_2Se_3 heterojunction

3. Temperature-dependent Characterization of Electrical Properties

4. Comparison of photoresponse in the visible range

5. Photocurrent mapping under 638 nm at different bias voltages

6. DFT calculation of individual Ta_2NiSe_5 and Bi_2Se_3 flakes

7. KPFM measurement and energy band diagrams of the Ta_2NiSe_5/Bi_2Se_3 heterojunction

8. Temporal photoresponse under various light powers

9. Energy band diagrams of the Bi_2Se_3/Ta_2NiSe_5 heterojunction at MWIR

10. Photoresponse and responsivity with bias voltage under different wavelengths and I-t curves under the illumination of 4.65 μm

11. Current noise power spectrum, EQE, and NEP of the heterojunction photodetector

12. Polarization characterization of different wavelengths

13. FTIR testing of silicon wafer

14. Comparison of the performance of our Ta_2NiSe_5/Bi_2Se_3 heterojunction photodetector with other relevant photodetectors

1. The schematic diagram of the PL optical measurement system

To characterize the electric properties of Ta_2NiSe_5/Bi_2Se_3 heterojunction, PL characterization was performed.

At roommate temperature, a 532 nm laser is used as a light source. The laser light is produced by adjusting the fiber optic demodulator, range of filters, and splitter orientation to ensure it is perpendicular to the photodetector. Through the focus of the objective lens, the light spot on the sample can be reduced to around 1 μm. The PL signal of the intended material area is analyzed by the spectrometer and the photoelectric signal can be obtained by the semiconductor film analyzer. Since the wavelength range of Ta_2NiSe_5 and Bi_2Se_3 photoluminescence is between 550 and 1000 nm, a filter with a wavelength of 500-1000 nm is used to filter the original laser signal.

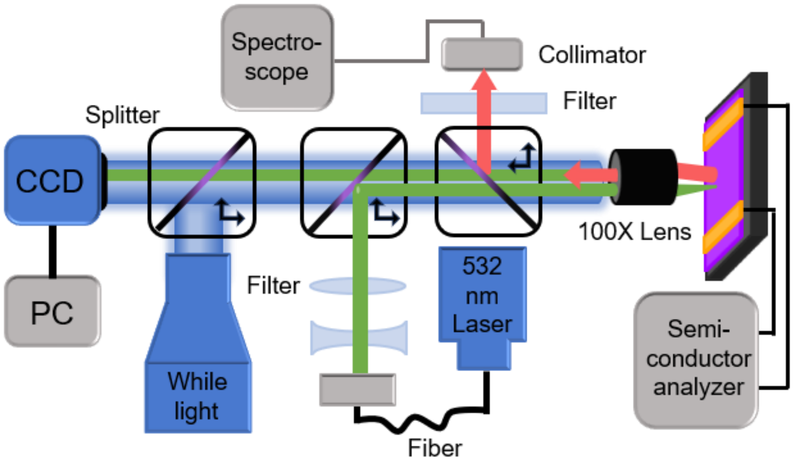

Figure S1 The schematic diagram of the PL optical measurement system.

2. The elemental composition analysis of the Ta_2NiSe_5/Bi_2Se_3 heterojunction

The distribution of elements in the analyzed area is determined by collecting and counting their characteristic X-rays using EDS under vacuum and electron beam bombardment. This allows for quantitative elemental analysis. The image on the monitor is formed by the brightness of the pixel points corresponding to the electron beam scanning of the specimen. The depth at which the characteristic X-rays escape is approximately 1000 nm. When expressing characteristic X-ray intensity in terms of brightness, the higher the brightness, the greater the content of the element. This is due to the varying layer spacing of atomic states for different elements, resulting in different excitation X-ray energies. The characteristic X-rays of Ta Mα1, Ni Lα1,2, Bi Mα1, and Se Lα1,2 elements were qualitatively collected from the SEM scanning region using an accelerating voltage of 10 keV. The atomic molar ratio of specific elements is shown in Figure S2.

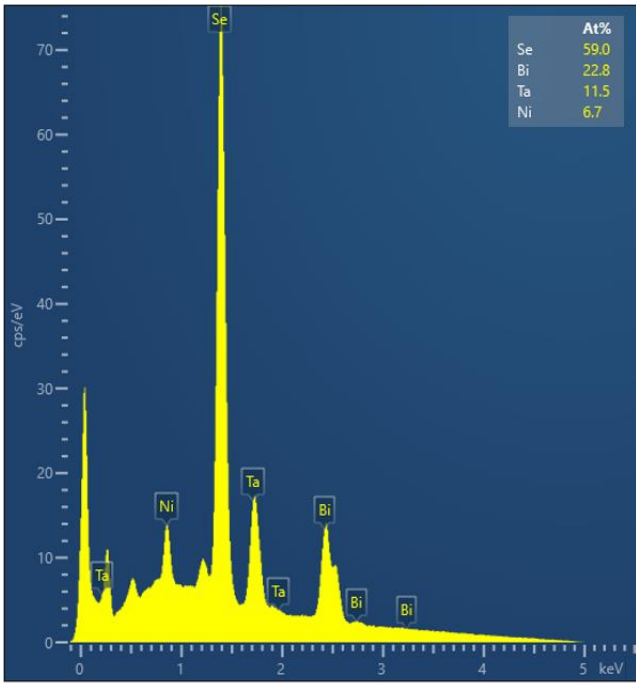

Figure S2. EDS spectrum of the Ta_2NiSe_5/Bi_2Se_3 heterojunction. The Ta: Ni: Bi: Se atomic molar ratio is summarized in the inset table.

3. Temperature-dependent Characterization of Electrical Properties

The heterojunction's resistance value exhibits a slow decreasing trend with increasing temperature, indicating that the photoinduced thermal effect can effectively enhance the photodetector conductance in Figure S3a. The suitability of the heterojunction for type-II energy band alignment is confirmed by fitting the equation in the semiconductor hot electron excitation model. In Figure S3c it can be seen that the heterojunction has a lower resistance value than Ta_2NiSe_5 and Bi_2Se_3 at room temperature, which also corresponds to the photoresponse in visible light below.

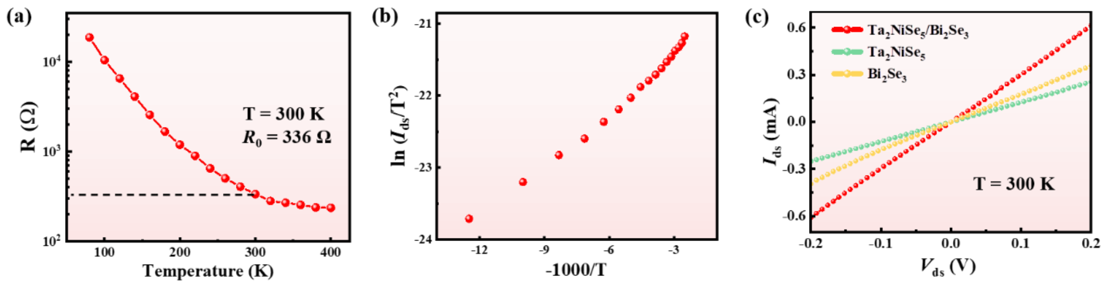

Figure S3. R-T characteristics, model fitted of the Ta_2NiSe_5/Bi_2Se_3 heterojunction and Comparison of I-V characteristics in darkness at room temperature. a) Resistance-Temperature characteristics of Ta_2NiSe_5/Bi_2Se_3 heterojunction in darkness. b) Thermionic emission model fitted results. The plots are extracted from Figure 2a at little forward bias. c) I_{ds}−V_{ds} curve of Ta_2NiSe_5, Bi_2Se_3 flakes, and the heterojunction photodetector in darkness at room temperature.

4. Comparison of photoresponse in the visible range

In Figure S4a-c, the result shows a stronger photoresponse for individual Bi_2Se_3 than for Ta_2NiSe_5 when the incident light power is varied. This corresponds to the results in Figure 2b of the main text. The heterojunction obtains the most pronounced photoresponse due to the type-II energy band alignment. Figure S4d demonstrates that the primary response mechanism of the heterojunction during visible light detection is the photovoltaic effect. The two-dimensional plot of photocurrent, which depends on the incident light power and bias voltage, can be divided into three parts: I, II, and III. These correspond to the photoresponse characteristics at different bias voltages, respectively.

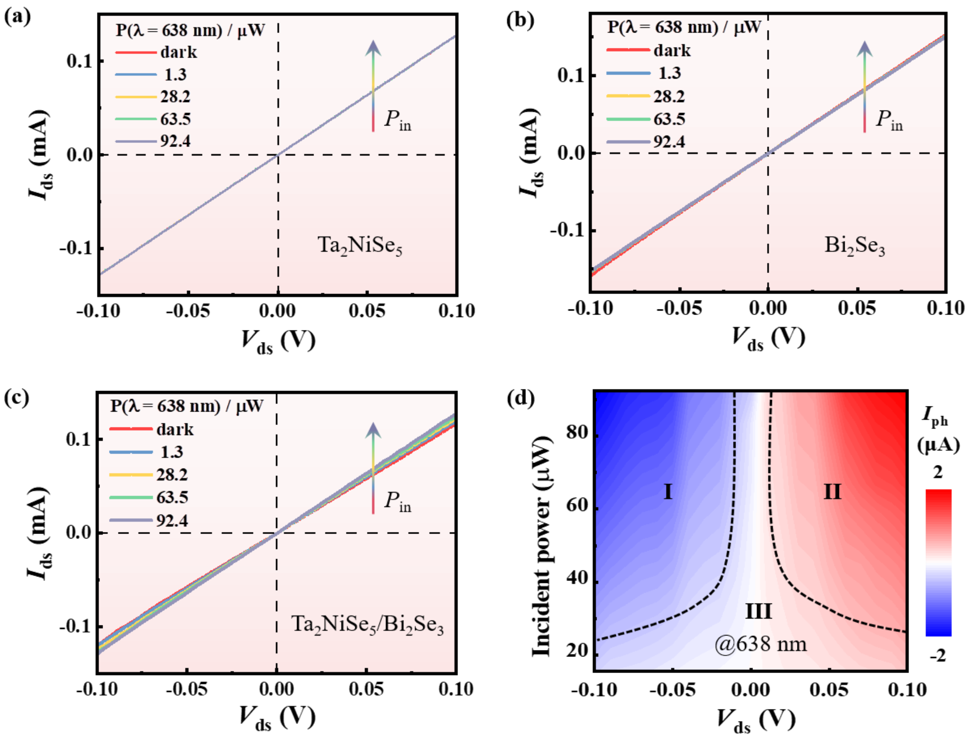

Figure S4. Comparison of I-V characteristics and Two-dimensional distribution diagram of photocurrent related to P_{light} and V_{ds} under 638 nm. a-c) I_{ds}–V_{ds} curve of Ta_2NiSe_5, Bi_2Se_3 flakes, and the heterojunction photodetector under 638 nm at various incident light powers. d) Incident power and V_{ds} dependent photocurrent extracted from the I-t curves with the value of the absolute.

5. Photocurrent mapping under 638 nm at different bias voltages

Furthermore, to precisely identify the location of photocurrent generation under visible light and confirm the photocurrent generation mechanism, we conducted a photocurrent mapping at various bias voltages. Figure S5a shows results similar to those in Figure 2c, where photocurrents are mainly generated in the overlapping region of the heterojunction.

Real-time imaging utilizes the differing reflectivity of the Au electrode, the SiO_2 substrate, and the material to determine the precise position of the Au electrode and the material. The yellow area in Figure S5b represents the unobstructed Au electrode, enabling accurate positioning of the photocurrent scanning area.

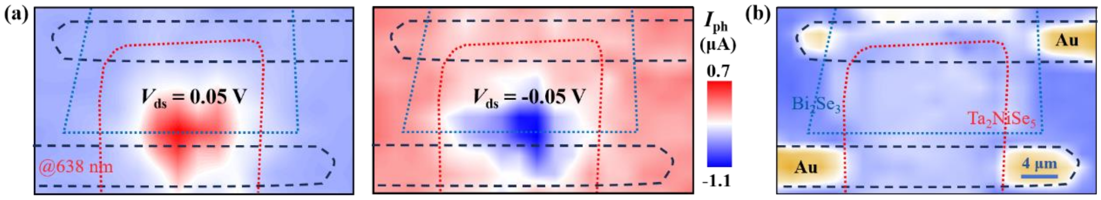

Figure S5. **Photocurrent mapping of heterojunction photodetectors at different bias voltages and corresponding real-time imaging map.** a) Photocurrent mapping images correspondingly measured at 0.05 V and -0.05 V bias under 638 nm laser illumination with P_{light} = 92.4 μW. b) Real-time photocurrent scanning map corresponding to a). The black dashed line is the Au electrode, the red dashed line is the Ta_2NiSe_5 and the blue dashed line is the Bi_2Se_3. Scale bar, 4 μm.

6. DFT calculation of individual Ta_2NiSe_5 and Bi_2Se_3 flakes

All calculations were performed using the Vienna ab initio simulation package (VASP) based on density functional theory (DFT) [1]. The exchange-correlation energy was treated within the generalized gradient approximation (GGA) framework using the Perdew–Burke–Ernzerhof (PBE) functional [2]. To obtain more precise band gap values, the Heyd–Scuseria–Ernzerhof (HSE06) method [3] was employed. The plane-wave cutoff energy was set to 400 eV, ensuring accurate description of electronic states. The sampling in reciprocal space was achieved using a 6×4×2 k-point mesh, enabling an appropriate representation of the Brillouin zone. The atomic structures were relaxed until the Hellmann-Feynman forces converged to less than 0.01 eV/Å, and the total energy reached convergence within 10-4 eV. These stringent convergence criteria ensure reliable and accurate results.

Ta_2NiSe_5 possesses a layered structure that is held together by weak van der Waals interactions. Within each layer, there are single chains of Ni atoms and double chains of Ta atoms running along the a-axis. The Se atoms are tetrahedrally coordinated around the Ni atoms and octahedrally coordinated around the Ta atoms (refer to Figure 1a). The valence band primarily consists of Ni-3d and Se-4p states with some contribution from Ta-5d states, while the conduction bands are mainly dominated by Ta-5d states with some contribution from Se-4p and Ni-3d states. It is worth noting that the valence and conduction bands in the orthorhombic phase belong to different irreducible representations of the crystal structure. This indicates distinct electronic properties and likely influences the material's overall behavior and performance. Further investigations and analysis are required to fully understand the implications of these band structures and their relationship to the physical properties of Ta_2NiSe_5. [4]

Figure 1a illustrates the atomic structure of Bi_2Se_3, which exhibits a rhombohedral crystal structure with the space group $R_3^{-}m$. It possesses layered structures with a triangular lattice. In Figure S6b, the band structure of Bi_2Se_3 indicates that it is a direct-

narrow gap semiconductor in its bulk form, with both the conduction band minimum (CBM) and valence band maximum (VBM) located at the Γ point. The valence band is primarily composed of Se-4p states with some contribution from Bi-6p states, while the conduction bands are mainly dominated by Se-4p and Bi-6p states.

By comparing the two figure parts, one can see clearly that the only qualitative change induced by turning on Spin-Orbit Coupling (SOC) is an anti-crossing feature around the 0 point, which thus indicates an inversion between the conduction band and valence band due to SOC effects, suggesting that Bi_2Se_3 is a topological insulator.

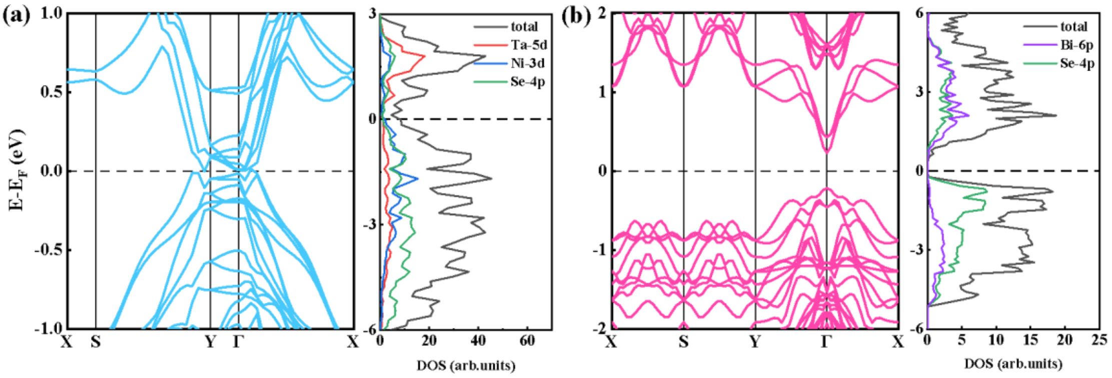

Figure S6. **Calculated band structure of Ta_2NiSe_5 and Bi_2Se_3 flakes.** Calculated band structure and Density of States (DOS) of a) flake Ta_2NiSe_5 and b) Bi_2Se_3 by DFT calculations. The dash line is the Fermi level which is set as 0.

7. KPFM measurement and energy band diagrams of the Ta_2NiSe_5/Bi_2Se_3 heterojunction

KPFM measurement was done to investigate band alignment of our Ta_2NiSe_5/Bi_2Se_3 heterojunction. Figure S7 shows a KPFM image of a typical Ta_2NiSe_5/Bi_2Se_3 heterojunction with contact potential difference ΔV_{CPD} between KPFM tip and the measured surface. Before measurement, the KPFM tip was calibrated by Au surface with work function around 5.1 eV, thus the work function of the tip was determined by $\Phi_{tip} = \Phi_{Au} + \Delta V_{CPD} = \Phi_{Au} + e \cdot \Delta CPD_{(tip\text{-}Au)}$ =5.1 eV-1.2 eV =3.9 eV. $\Delta CPD_{(tip\text{-}Au)}$ is the difference of CPD between tip and Au which is equal to the difference of surface potentials between tip and Au. Therefore, the work functions of Bi_2Se_3 and Ta_2NiSe_5 estimated by $\Phi_{sample} = \Phi_{tip} - \Delta V_{CPD} = \Phi_{tip} - e \cdot \Delta CPD_{(tip\text{-}sample)}$ can be derived from the KPFM measurement($\Delta CPD_{(tip\text{-}Bi2Se3)}$ = -0.7 eV, $\Delta CPD_{(tip\text{-}Ta2NiSe5)}$ = -0.91 eV), which is ~ 4.6 eV and ~ 4.81 eV, respectively.

Under different bias conditions, as shown in Figure S7b, at forward bias, the external bias voltage will break the unified Fermi level, simultaneously enhancing the built-in electric field. Consequently, the holes in Ta_2NiSe_5 can easily cross the barrier and reach the Bi_2Se_3 side to form a significant current, which generates the current-rectifying characteristics of the heterojunction. Meanwhile, when the photodetector operates under a reverse bias, the diminished built-in potential barrier finds it hard to block the transfer of electrons from Ta_2NiSe_5 to Bi_2Se_3, which results in the conduction state occurring at the reverse bias.

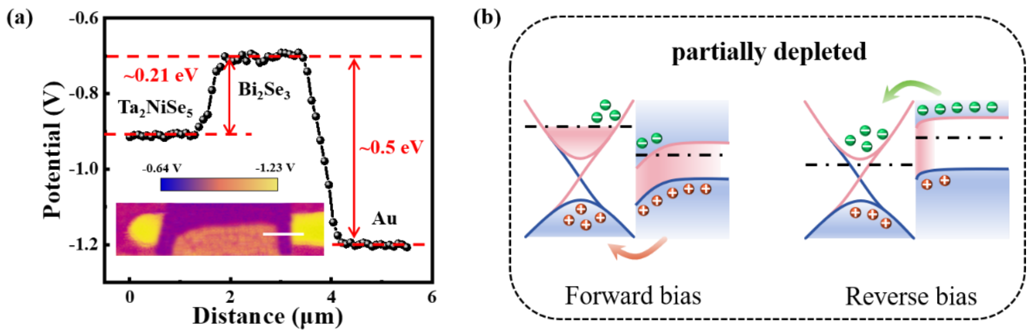

Figure S7. **KPFM potential difference measurements and corresponding energy band diagram alignments of the Ta_2NiSe_5/Bi_2Se_3 heterojunction.** a) KPFM measurement of the potential difference between Ta_2NiSe_5/Bi_2Se_3 heterojunction and Au electrode along the white line. The inset image is the KPFM measurement mapping of the Ta_2NiSe_5/Bi_2Se_3 heterojunction photodetector. b) Energy band alignment diagrams of Bi_2Se_3/Ta_2NiSe_5 heterojunction with forward and reverse bias voltage.

8. Temporal photoresponse under various light powers

Figures S8a-f demonstrate that the heterojunction photodetector exhibits stable and rapid photocurrent switching characteristics when exposed to 520, 638, 940, 1064, 1310, and 1650 nm lasers. This is supported by the photocurrent response speed shown in Figure S10a.

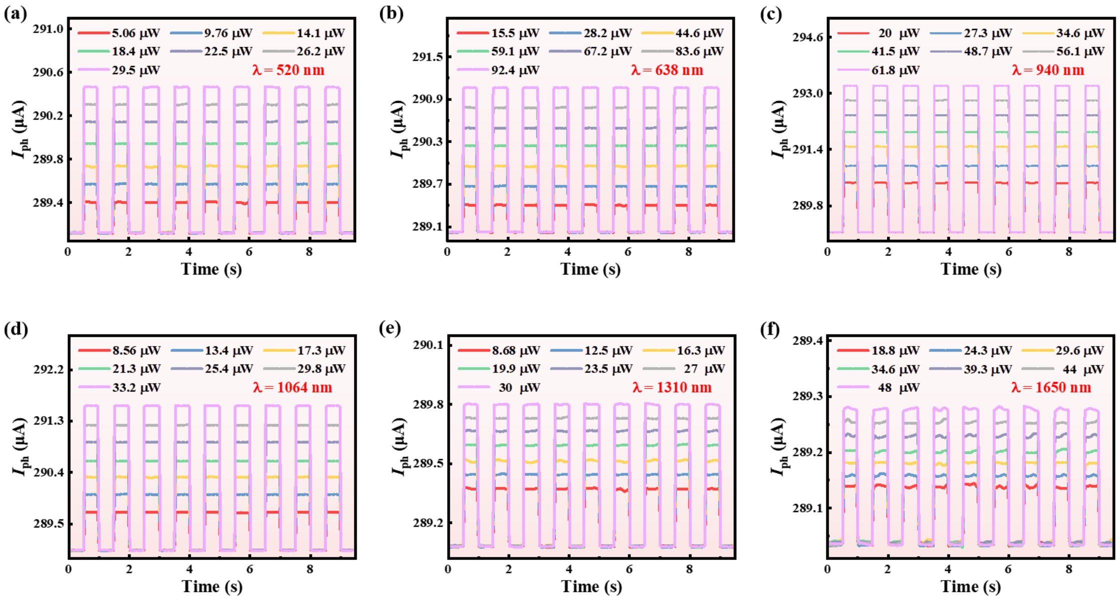

Figure S8. The incident light power dependence response of the Ta_2NiSe_5/Bi_2Se_3 photodetector from 520 to 1650 nm. a-f) The temporal photoresponse of Ta_2NiSe_5/Bi_2Se_3 heterojunction photodetector from 520 nm to 1650 nm under various incident light powers. All measurements were carried out in ambient air tested by our source meter (Keithley 6482) at a modulation frequency of 1 Hz.

9. Energy band diagrams of the Bi_2Se_3/Ta_2NiSe_5 heterojunction at MWIR.

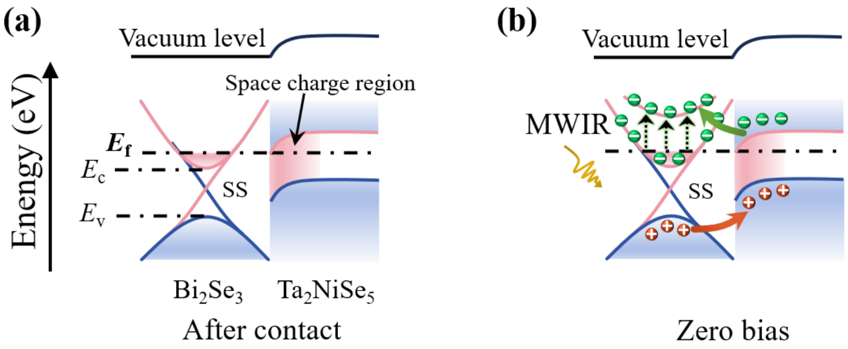

Figure S9. Energy-band diagrams and microscopic mechanisms of photocurrent generation for Bi_2Se_3/Ta_2NiSe_5 heterostructure photodetector at MWIR. a) Energy-band diagram of the Bi_2Se_3/Ta_2NiSe_5 heterostructure at thermal equilibrium status in the dark. SS is the surface state of the Bi_2Se_3 topological insulator film. E_c and E_v denote the conduction-band bottom and valence-band top, respectively, and E_f is the Fermi level. The width of space charge region is not scaled. b) Working mechanism of the Bi_2Se_3/Ta_2NiSe_5 heterostructure photodetector at zero bias voltage. Vertical arrow represents photoexitation, and lateral arrows indicate the transport directions of electrons and holes contributing to the photocurrent.

10. Photoresponse and responsivity with bias voltage under different wavelengths and I-t curves under the illumination of 4.65 μm.

Furthermore, Figure S10a demonstrates that the photocurrents of the heterojunction photodetector display comparable intensity trends when the polarity of the positive and negative bias voltages changes at various laser irradiation wavelengths (638 nm, 1550 nm, and 4.65 μm). This finding strongly confirms that the photovoltaic photoresponse mechanism is affected over a wide band. In Figure S10c, the optical response at 4.65 μm exhibits a slower increasing trend compared to the visible and near-infrared bands. This suggests a slower response due to the influence of the defect states in the photogating effect on the carrier-separated transport.

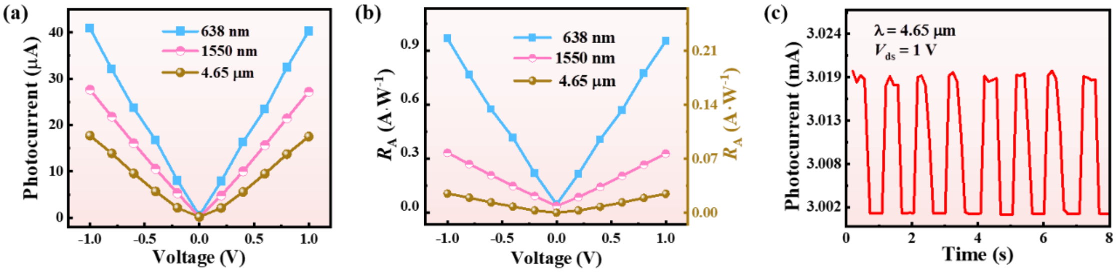

Figure S10. ***I_{ph}*-*V_{ds}* characterizations and responsivity of the photodetector under different wavelengths and *I_{ph}*-t curves with 1 V bias under 4.65 μm.** a) Photocurrents of the heterojunction photodetector were measured at different wavelengths while varying the bias from -1 to 1 V. b) R_A with bias voltage corresponding to different wavelengths c) Photoswitching response of the Ta_2NiSe_5/Bi_2Se_3 heterojunction photodetector under 4.65 μm laser illumination with P_{light} = 57 μW (The modulation frequency is 1 Hz).

11. Current noise power spectrum, EQE, and NEP of the heterojunction photodetector

The response time and the noise power spectra $S_n(f)$ of the heterojunction photodetector were analyzed by using an N9010B signal analyzer (Keysight EXA Signal Analyzer) and an SR570 current preamplifier. The photodetector was enclosed in a metal casing to protect it from surrounding noise.

From the current noise power spectrum, the low-frequency (10 Hz-1 kHz) noise power contribution is dominated by 1/f noise, which originates from the fluctuation of carriers being trapped and de-trapped by the defect centers. Above 1 kHz, the current noise power density becomes independent of frequency, resulting in white noise. Shot noise and thermal noise are two types of white noise that may dominate the noise contribution in the higher frequency range.

The EQE and NEP can be calculated from the following equation: EQE = $hcR_A/e\lambda$, NEP = i_n/R_A, where h is 6.626×10^{-34}, c is 3×10^{8} m/s, e is 1.6×10^{-19} C and $i_n = (i_t^2+i_s^2)^{1/2} = (9.62^2+7.02^2)^{1/2} = 11.9$ pA·Hz$^{-1/2}$. Figure S11b shows that both sensitivity performance parameters have the same trend with response wavelength as the photoresponse.

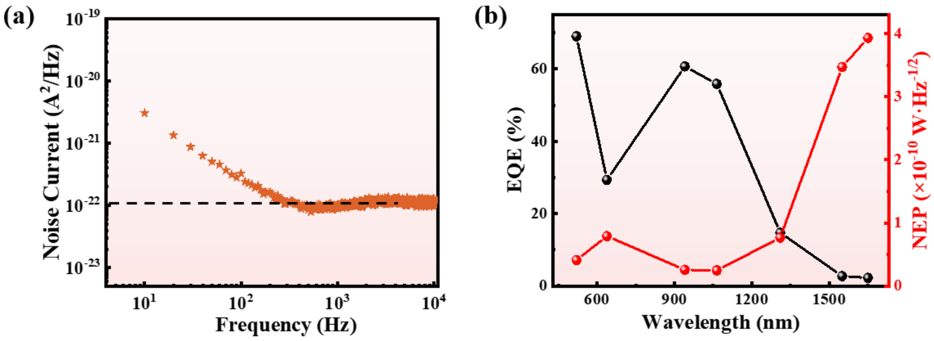

Figure S11 Current noise power spectrum of the heterojunction photodetector and curves of their performance parameters EQE and NEP as a function of wavelength. a) The current noise power spectrum with frequencies ranging from 10 Hz to 10 kHz. It is suitable for the device of 520 nm to 4.65 μm. b) EQE and NEP of the heterojunction photodetector with different wavelengths from 520-1650 nm.

12. Polarization characterization of different wavelengths

The function that fits the photocurrent (I_{ph}) response to the angle of polarization (θ) of the incident light is $I_{ph}(\theta)=I_{py}\cdot\cos^2(\theta+\varphi)+I_{px}\cdot\sin^2(\theta+\varphi)$. This function is based on the concept of anisotropic response. By fitting the experimental data of the extracted photocurrent to this formula and determining the values of I_{py} and I_{px}, the amplitudes of the photocurrent for the two orthogonal polarization directions can be obtained. This fitting process allows for quantifying the anisotropic ratio and the polarization-dependent behavior of the device. The directional dependence of the photocurrent response in the photodetector is determined by obtaining the specific anisotropy ratio index and polarization-related phenomena. The heterojunction photodetector exhibits polarization characteristics over a wide spectrum, indicating potential for further applications such as optical switching, navigation, and high-contrast polarizers.

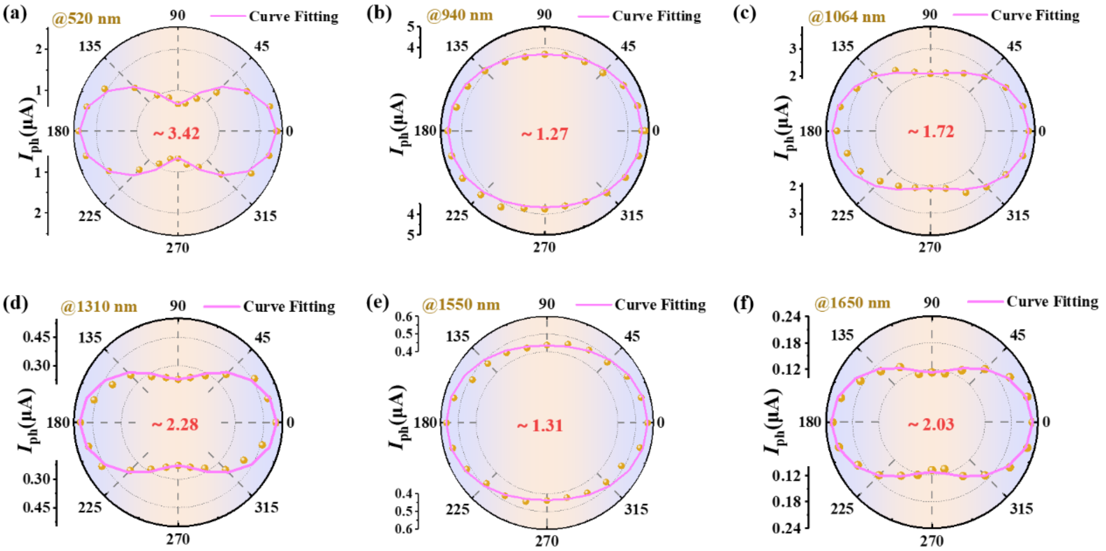

Figure S12 Polarization characterization over a broad spectrum. a-f) Polar plots illustrate the angularly resolved photocurrents generated by irradiation with a linear-polarization laser within the range of 520-1650 nm. The polarization ratios at different wavelengths were provided in the middle numbers.

13. FTIR testing of silicon wafer

Figure S13 shows the results of the FTIR from visible to near-infrared transmittance test performed on the double-thrown low-resistance silicon wafer.

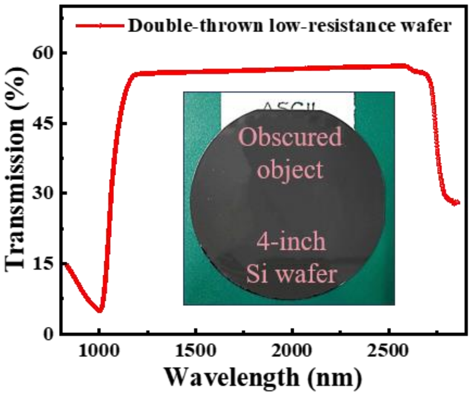

Figure S13 FTIR testing of silicon wafer comparing transmittance in the visible and near-infrared bands. The embedded figure illustrates the physical view of the silicon wafer used for dual-channel imaging.

14. Table S1. Comparison of the Performance of Our Ta_2NiSe_5/Bi_2Se_3 Heterojunction Photodetector with Other Relevant Photodetectors.

Structures	Wavelength (nm)	R_A ($A \cdot W^{-1}$)	D^* ($cm \cdot Hz^{1/2} \cdot W^{-1}$)	Response time	Bias voltage (V)	polarization ratio	Ref.
Bi_2Se_3	1456	2.74	3.3×10^{10}	540/470 ms	1	-	[5]
Bi_2Te_3	1064–1550	3.64×10^{-3} (1064 nm)	-	-	2	-	[6]
Ta_2NiSe_5	520-4600	0.86 (4600 nm)	8.75×10^{8}	24/26 ms	1	1.47	[7]
Bi_2Se_3/SnTe	1550	0.146	1.15×10^{10}	6.9/19.2 µs	0	-	[8]
Bi_2Se_3/Bi_2O_2Se	532-1450	42.9×10^{-6} (850 nm)	1.18×10^{7}	274/318 ms	0	-	[9]
Bi_2Se_3/WSe_2	532/1456	3 (1456 nm)	2.2×10^{10}	4 ms	-5	-	[10]
Bi_2Se_3/graphene	520	1.4	-	114.94 ms	1.5	-	[11]
Bi_2Te_3/WS_2	1550	1.9×10^{-3}	2.7×10^{7}	20 ms	1	-	[12]
Bi_2Te_3/WSe_2	1550	27×10^{-3}	-	-	1	-	[13]
$WSe_2/Ta_2NiSe_5/WSe_2$	400–1550	0.436 (635 nm)	1×10^{12}	420/640 µs	0	14.8	[14]
Ta_2NiSe_5/WSe_2	638/1550	0.82×10^{-9} (1550 nm)	90	278/283 µs	0	-	[15]
Ta_2NiSe_5/MoS_2	532/1064	0.7 (1064 nm)	2.4×10^{9}	7.4/31.1 s	1	-	[16]
Ta_2NiSe_5/GaSe	520/1550	0.15 (1550 nm)	1.08×10^{9}	370/540 ms	-3	-	[17]
BP/InSe	455	11.7×10^{-3}	-	24 ms	0.5	0.83	[18]
WS_2/ReS_2	532	1.85×10^{-3}	-	1.3 ms	1.5	1.85	[19]
This work	520-4650	0.46 (1064 nm)	3.8×10^{11}	168/151 µs	0.1	3.42 (520 nm)	

References:

[1] G. Kresse, J. Furthmüller, *Phys. Rev. B* **1996**, *54* (16), 11169.

[2] J. P. Perdew, K. Burke, M. Ernzerhof, *Phys. Rev. Lett.* **1996**, *77* (18), 3865.

[3] J. Heyd, G. E. Scuseria, M. Ernzerhof, *J. Chem. Phys.* **2003**, *118* (18), 8207.

[4] Y. Lu, H. Kono, T. Larkin, A. Rost, T. Takayama, A. Boris, B. Keimer, H. Takagi, *Nat. Commun.* **2017**, *8* (1), 14408.

[5] F. Wang, L. Li, W. Huang, L. Li, B. Jin, H. Li, T. Zhai, *Adv. Funct. Mater.* **2018**, *28* (33), 1802707.

[6] J. Liu, Y. Li, Y. Song, Y. Ma, Q. Chen, Z. Zhu, P. Lu, S. Wang, *Appl. Phys. Lett.* **2017**, *110* (14).

[7] S. Zhang, L. Han, K. Xiao, L. Zhang, C. Shi, L. Xu, K. Deng, Y. Zou, M. Jiang, X. Lv, *Adv. Funct. Mater.* **2023**, *33* (48), 2305380.

[8] H. Zhang, Z. Song, D. Li, Y. Xu, J. Li, C. Bai, B. Man, *Appl. Surf. Sci.* **2020**, *509*, 145290.

[9] M. Yu, C. Fang, J. Han, W. Liu, S. Gao, K. Huang, *ACS Appl. Mater. Interfaces* **2022**, *14* (11), 13507.

[10] F. Wang, P. Luo, Y. Zhang, Y. Huang, Q. Zhang, Y. Li, T. Zhai, *Sci. China Mater.* **2020**, *63* (8), 1537.

[11] J. Chae, S.-B. Hong, D. Kim, D.-K. Kim, J. Kim, K. Jeong, S. H. Park, M.-H. Cho, *Appl. Surf. Sci.* **2021**, *554*, 149623.

[12] J. Yao, Z. Zheng, G. Yang, *J. Mater. Chem. C* **2016**, *4* (33), 7831.

[13] H. Liu, X. Zhu, X. Sun, C. Zhu, W. Huang, X. Zhang, B. Zheng, Z. Zou, Z. Luo, X. Wang, *ACS Nano* **2019**, *13* (11), 13573.

[14] T. Zheng, M. Yang, Y. Pan, Z. Zheng, Y. Sun, L. Li, N. Huo, D. Luo, W. Gao, J. Li, *ACS Appl. Mater. Interfaces* **2023**, *15* (24), 29363.

[15] P. Xiao, S. Zhang, L. Zhang, J. Yang, C. Shi, L. Han, W. Tang, B. Zhu, *Sensors* **2023**, *23* (9), 4385.

[16] T. Guo, X. Song, P. Wei, J. Li, Y. Gao, Z. Cheng, W. Zhou, Y. Gu, X. Chen, H. Zeng, *ACS Appl. Mater. Interfaces* **2022**, *14* (50), 56384.

[17] Y. Zhang, L. Huang, J. Li, Z. Dong, Q. Yu, T. Lei, C. Chen, L. Yang, Y. Dai, J. Zhang, *Appl. Phys. Lett.* **2022**, *120* (26)), 261101.

[18] S. Zhao, J. Wu, K. Jin, H. Ding, T. Li, C. Wu, N. Pan, X. Wang, *Adv. Funct. Mater.* **2018**, *28* (34), 1802011.

[19] Y. Tang, H. Hao, Y. Kang, Q. Liu, Y. Sui, K. Wei, X. a. Cheng, T. Jiang, *ACS Appl. Mater. Interfaces* **2020**, *12* (47), 53475.
